# The role of procalcitonin in differential diagnosis between acute radiation pneumonitis and bacterial pneumonia in lung cancer patients receiving thoracic radiotherapy

**DOI:** 10.1038/s41598-020-60063-w

**Published:** 2020-02-19

**Authors:** Zhiwu Wang, Bingjie Huo, Qiong Wu, Liang Dong, Haoyu Fu, Shuo Wang, Jing Zhang

**Affiliations:** 1 Department of Chemoradiotherapy, Tangshan People’s Hospital, Tangshan, P.R. China; 2 Department of traditional Chinese medicine, The Fourth Hospital of Hebei Medical University, Shijiazhuang, P.R. China; 3 Department of Radiology, Tangshan People’s Hospital, Tangshan, P.R. China; 4 Department of Endocrinology, Tangshan People’s Hospital, Tangshan, P.R. China

**Keywords:** Biomarkers, Diseases, Oncology

## Abstract

Acute Radiation Pneumonitis (ARP) is one of the most common dose-limiting toxicities of thoracic radiotherapy. The accurate diagnosis of ARP remains a challenge because of the lack of a rapid biomarker capable of differentiating ARP from bacterial pneumo (BP). The aim of this study was to investigate the potential usefulness of procalcitonin (PCT) in the differential diagnosis of ARP and BP. Lung cancer patients who had undergone thoracic radiotherapy within 6 months and were admitted to hospital for ARP or BP were retrospectively analyzed. The serum levels of PCT, C-reactive protein (CRP) and white blood cells (WBC) were compared between the two groups. Receiver operating characteristic (ROC) curve was used to assess the diagnostic value of PCT, CRP and WBC in the differential diagnosis of ARP and BP and determine the best cut-off values. One hundred eighteen patients were included. Among them, seventy-seven patients were diagnosed with ARP, and 41 patients were diagnosed with BP. The PCT concentrations for patients diagnosed with ARP group were significantly lower than those in the BP group (P < 0.001). There were no differences in CRP and WBC between the two groups. The areas under the ROC curves (AUC) for PCT, CRP and WBC were 0.745, 0.589 and 0.578, respectively. The best cutoff values of PCT, CRP and WBC were 0.47 μg/L, 54.5 mg/L and 9.9 × 10^9^/L, respectively. Low serum PCT levels are associated with ARP. PCT is a useful biomarker to distinguish ARP from BP.

## Introduction

Radiotherapy has been widely used for the curative and palliative treatment of lung cancer. Radiation-induced lung injury (RILI) is the most common dose-limiting toxicity of thoracic radiotherapy^1^. RILI includes acute radiation pneumonitis (ARP) and pulmonary fibrosis. ARP often occurs about 1 to several months after radiotherapy. Commonly, ARP is relatively mild with patients, exhibiting no obvious symptoms except for imaging abnormalities. However, ARP can be a very serious event, especially after large volume radiotherapy. The incidence of grade 3–5 ARP can reach about 20% according to a recent study^2^. When symptoms are more severe, patients cannot be managed as outpatients and require hospitalization. Continued progression of symptomatic ARP can cause respiratory failure and even death. Early and timely intervention can prevent the progression of ARP, improve patient symptoms, and potentially reduce post-radiation pulmonary fibrosis.

However, accurate diagnosis of ARP remains difficult. The most important issue for radiation oncologists is to differentiate them from the bacterial pneumonia (BP) which are common in lung cancer patients^3^. ARP and BP share many of the same symptoms such as cough and fever and both can be identified by changes in imaging. ARP can usually be identified by the positional relationship between the shapes of the radiation field and the inflammatory lesion. However, the clinical judgment is affected by the extensive application of intensity modulation technology for lung cancer which increases the area of normal lung exposure. Bacterial culture is an accurate method to help the judgment, but requires significant time. Therefore, it is necessary to explore diagnostic biomarkers with higher sensitivity and specificity for distinguishing symptomatic ARP and BP.

Procalcitonin (PCT), as a calcitonin precursor, is involved in the systemic reaction induced by the circulating endotoxins and inflammatory cytokines^4,5^. Several studies showed PCT serum level is significantly higher in patients with bacterial infections, such as pneumonia, than in those with viral infections and non-infectious diseases and is used as a diagnostic marker for bacterial infections^6^. Therefore, our study investigates the prospect of using PCT as a critical biomarker for making a differential diagnosis between ARP and BP.

## Materials and Methods

### Study population

This retrospective study included patients who were hospitalized in the department of chemoradiotherapy, Tangshan People’s Hospital, from January 2014 to December 2016. The eligibility criteria were as follows: (1) patients had been histologically or clinically diagnosed with lung cancer; (2) patients had received thoracic radiotherapy within 6 months; (3) patients had acute pneumonia symptoms including fever, cough and dyspnea etc.; (4) patients had pulmonary inflammatory lesions on computed tomography (CT) images; (5) PCT test was performed immediately after admission. Exclusion criteria: patients had the symptoms and signs in the inclusion criteria caused by the following factors: tumor progression, acute exacerbation of chronic obstructive pulmonary disease (COPD), cardiogenic disease, pulmonary infarction, anemia, etc.

### Diagnostic criteria

Diagnostic criteria for BP: On the basis of satisfying the above conditions (except for item 5), BP needs to be confirmed by microbiological examination which included blood culture and sputum culture (limited to high-quality specimens, defined as ≤10 epithelial cells and ≥25 white blood cells per low-power field). Diagnostic criteria for ARP: Patients meet the above inclusion criteria, except for the factors related to infectious pneumonia; Glucocorticoid therapy is effective; CT imaging changes are mainly limited to the radiation field.

### Information collection and adjudication

There were two groups responsible for the information collection and adjudication. The information of the patients was collected from medical records by one group (Qiong Wu, Liang Dong and Haoyu Fu) and was submitted to the adjudication group (Zhiwu Wang, Zhang Jing and Shuo Wang). The physicians in charge of adjudication were blind for the biomarkers results. Every case was reviewed independently by three physicians of the adjudication group and differences were resolved by consensus. During the review, adjudicators should answer at least three questions: 1 Did the pathogenic microbiological examination suggeste a bacterial infection? 2 Were CT imaging changes mainly distributed in the radiation field? 3 Was glucocorticoid treatment effective? Adjudication works were based on the inclusion criteria, exclusion criteria, and diagnostic criteria mentioned above.

### Laboratory assessment

Blood samples (3 mL per person) were drawn from peripheral veins immediately after admission and stored at 4 °C. Testing was carried out within 3 hours after blood was draw. PCT level was determined by the Roche Elecsys Brahms procalcitonin assay (Roche Diagnostics GmbH, Germany), which was performed on a Cobas e601 analyzer (Roche Diagnostics GmbH, Germany). CRP level was measured by immunoturbidimetry. The number of white blood cells (WBC) was also recorded at the same time.

### Statistical analysis

Continuous variables were analyzed with Student t-test. Categorical variables were examined using x2-test. All tests were two-sided and p < 0.05 was taken to indicate statistical significance. The areas under the receiver operating characteristic (ROC) curves were calculated for the PCT, CRP, and WBC as predictors of ARP and cut-off with the highest Youden’s Index value was chosen for each inflammatory marker. Analysis was performed with the SPSS statistical software package (SPSS, Inc., Chicago, IL, USA), version 19.0.

### Ethics statement

This study was approved by ethics committee of Tangshan People’s Hospital and all methods were carried out in accordance with the ethical rules of the Helsinki Declaration and Good Clinical Practice. Written informed consent was waived by the ethics committee of Tangshan People’s Hospital because this was a retrospective, non-invasive and observational study.

## Results

**Figure 1:**
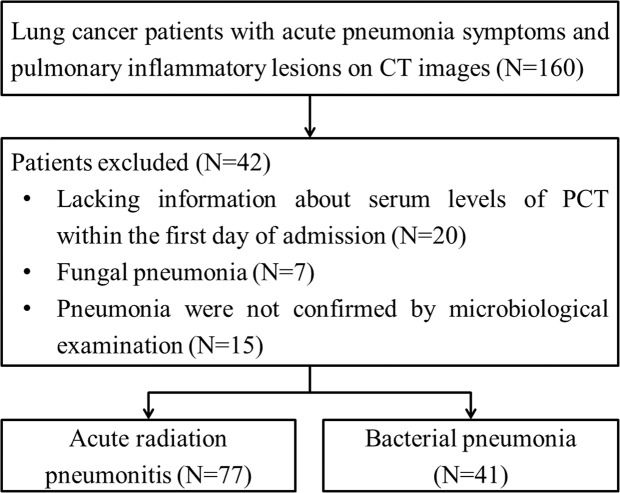
Flow diagram of the retrospective enrollment in the study.

Among 160 consecutive patients being screened, 118 patients were included in this study and 42 were excluded (Fig. 1). The baseline characteristics of the included patients are presented in Table 1. Of the 118 patients, there were 85 (72.0%) males and 33 (28.0%) females, with a median age of 61 years (range 36–81). The highest proportion of pathological type was adenocarcinoma (33.9%), followed by small cell carcinoma (32.2%) and squamous cell carcinoma (28.8%). Non-small cell lung cancer not otherwise specified (NSCLC, NOS) (5.1%) comprised the remainder. Patients were staged according to the American Joint Committee on Cancer 2010 guidelines^7^. There were 78 (66.1%) patients with stage II and III and 40 (33.9%) with stage IV. Concerning the physical status, 71 (60.2%) patients had an Eastern Cooperative Oncology Group Performance Status Scale (ECOG PS) of 0–1 and the remaining 47 (39.8%) patients had a PS score of 2–3.

**Table 1:** Clinical Characteristics of all patients and the patients in the ARP and BP groups.

Characteristic	Overall	ARP	BP	*P*
No. patients	118	77	41	
Age (%)				0.243
≥65	49 (41.5)	29 (37.7)	20 (48.8)	
<65	69 (58.5)	48 (62.3)	21 (51.2)	
Sex (%)				0.528
Male	85 (72.0)	54 (70.1)	31 (75.6)	
Female	33 (28.0)	23 (29.9)	10 (24.4)	
Histopathology(%)				0.859
Adenocarcinoma	40 (33.9)	26 (33.8)	14 (34.1)	
Squamous cell	34 (28.8)	21 (27.3)	13 (31.7)	
SCLC	38 (32.2)	25 (32.5)	13 (31.7)	
NSCLC, NOS	6 (5.1)	5 (6.5)	1 (2.4)	
Stage (%)				0.391
II–III	78 (66.1)	53 (68.8)	25 (61.0)	
IV	40 (33.9)	24 (31.2)	16 (39.0)	
PS(%)				0.510
≤1	71 (60.2)	48 (62.3)	23 (56.1)	
2–3	47 (39.8)	29 (37.7)	18 (43.9)	
Radiation dose, (%)				0.016
≥62Gy	30 (25.4)	25 (32.5)	5 (12.2)	
<62Gy	88 (74.6)	52 (67.5)	36 (87.8)	
MLD (%)				0.017
≤17.4Gy	42 (35.6)	33 (42.9)	9 (22.0)	
>17.4Gy	76 (64. 4)	44 (57.1)	32 (78.0)	
V20 (%)				0.235
≤30%	49 (41.5)	35 (45.5)	14 (34.1)	
>30%	69 (58.5)	42 (54.5)	27 (65.9)	

*ARP* acute radiation pneumonitis, *BP* bacterial pneumonia; *MLD* mean lung dose; *V20* volume of total lung exceeding 20 Gy.

A total of 77 patients were diagnosed with ARP, and the remaining 41 ones were diagnosed with BP. No significant difference was observed between the ARP and BP groups in age, sex, PS score or tumor stages. However, there was a significant difference in the radiation dose parameters. Patients in the APR group received higher prescription dose and mean lung dose (MLD) than these in the BP group, but there was no significant difference in percentage volume of total lung exceeding 20 Gy (V20) between the two groups.

**Figure 2:**
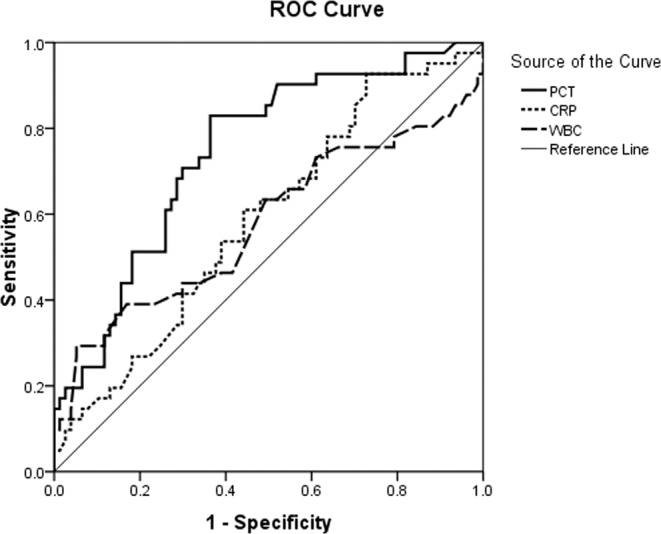
ROC curves of PCT, CRP and WBC.

As shown in Table 2, the median PCT concentration was 0.50 μg/L (Interquartile Range: 0.34–1.12). The PCT concentrations in the ARP group were significantly lower than those in the BP group (P < 0.001). The median CRP concentration and WBC count were 97.6 mg/L (Interquartile Range: 62.0–129.3) and 7.3 × 10^9^/L (Interquartile Range: 5.9–8.6), respectively. There were no differences between the groups in CRP and WBC. The areas under the ROC curves (AUC) for PCT, CRP and WBC were 0.745 (95% CI: 0.653–0.836; P < 0.001), 0.589 (95% CI: 0.482–0.695; P = 0.114) and 0.578 (95% CI: 0.460–0.696; P = 0.164), respectively (Fig. 2). The best cutoff values of PCT, CRP and WBC established by ROC were 0.47 μg/L, 54.5 mg/L and 9.9 × 10^9^/L, respectively. With these cutoffs, the sensitivity, specificity, positive predictive value (PPV), negative predictive value (NPV), false positive rate (FPR), and false negative rate (FNR) for the detection of ARP were obtained (Table 3).

**Table 2:** Levels (Medians and Interquartilic Ranges) of PCT, CRP and WBC in all patients and the patients in the ARP and BP groups.

Variable	Overall	ARP	BP	*P*
PCT	0.50 (0.34–1.12)	0.41 (0.29–0.85)	0.97 (0.50–1.57)	0.000
CRP	97.6 (62.0–129.3)	87.3 (53.0–127.0)	104.2 (73.3–135.9)	0.114
WBC	7.3 (5.9–8.6)	7.2 (5.9–8.2)	7.3 (6.2–10.2)	0.163

*CRP* C-reactive Protein; *PCT* Procalcitonin; *WBC* White blood cell; See Table 1 for other abbreviations.

**Table 3:** Sensitivity, Specificity, PPV and NPV of PCT, CRP and WBC as diagnostic indicators of ARP.

Variable	Sensitivity, % (95% CI)	Specificity, % (95% CI)	PPV, % (95% CI)	NPV, % (95% CI)	FPR, % (95% CI)	FNR, % (95% CI)
PCT	63.6 (51.9–74.3)	82.9 (67.9–92.8)	87.5 (75.2–95.1)	54.8 (42.9–65.8)	17.1 (7.2–32.1)	36.4 (25.7–48.1)
CRP	27.3 (17.7–38.6)	92.7 (80.1–98.5)	87.5 (62.5–98.0)	40.4 (34.1–46.1)	7.32 (1.5–19.9)	72.7 (61.4–82.3)
WBC	94.8 (87.2–98.6)	29.3 (16.1–45.5)	71.6 (66.1–77.3)	75.0 (40.1–94.5)	70.7 (54.5–83.9)	5.2 (1.4–12.8)

*NPV* negative predictive value; *PPV* positive predictive value; *FPR* false positive rate; *FNR* false negative rate; See Tables 1 and 2 for other abbreviations.

The bacterial pathogens of 38 BP patients (92.7%) were detected by sputum culture and 3 cases (7.3%) were confirmed by blood culture. Most agents isolated were gram-negative germs (n = 33, 80.5%) among which *Klebsiella pneumonia* and *Pseudomonas aeruginosa* accounted for 43.9% (n = 18). The detailed microbiology results are presented in Table 4.

**Table 4:** Microbiology Results in the Bacterial Pneumonia Group.

Microorganism	No. Patients (%)
Gram-Negative germs	33 (80.5)
Klebsiella pneumoniae	11 (26.8)
Pseudomonas aeruginosa	8 (19.5)
Acinetobacter baumannii	5 (12.2)
Enterobacter cloacae	5 (12.2)
Haemophilus influenzae	2 (4.9)
Stenotrophomonas maltophilia	2 (4.9)
Gram-Positive germs	8 (19.5)
Aureus	5 (12.2)
Staphylococcus epidermidis	3 (7.3)

## Discussion

This retrospective study comprised of 118 lung cancer patients who were admitted to the hospital for pneumonia and had undergone thoracic radiotherapy within 6 months, including 77 ARP and 41 BP patients, found the PCT levels in the ARP group to be significantly lower than those in the BP group (P < 0.001). The two groups were determined not to differ in CRP and WBC. The AUC for PCT, CRP and WBC were 0.745, 0.589 and 0.578, respectively. The best cutoff value of PCT was 0.47 μg/L. Based on the above results, PCT could be a useful biomarker to distinguish ARP from BP.

PCT is a useful biomarker for the diagnosis and monitoring of bacterial infections^8^ which is widely used in clinic. It should also help physicians to identify non-infectious diseases from infectious diseases. A study demonstrated PCT was valuable in determining the cause of febrile episodes in patients with advanced urological cancer^9^. Another study found that patients with non-infections fever had lower PCT levels than those with infectious fever^10^. Considering that ARP is essentially non-infectious inflammation which is similar to the non-infectious fever in the study mentioned above, we speculated that PCT should also play a role in distinguishing ARP from BP. The result of the present study confirmed our hypothesis. The result of a previous study in which the authors established a scoring system to differentiate ARP from BP using PCT as one of the classifying factors^11^ is consistent with and indirectly supports the finding in this work.

The application of PCT in patients with lung cancer remains controversial. In a previous study, the researchers compared serum PCT levels in infected and non-infected lung cancer patients and found no difference between the two groups^12^. The study also found no difference in CRP levels between the two groups, while there was a strong correlation between PCT and CRP (r = 0.80). Our study also found a correlation between PCT and CRP, but the PCT has been shown to be useful for distinguishing between ARP and BP. The reason for the inconsistent results remains unclear. A firm understanding of the contradictory results may be achieved with further prospective studies.

The false-positive rate of PCT in this study was 17.78%. The reason is not exclusive of the increase in PCT level caused by lung cancer itself, but it is unclear how much the lung cancer itself affects the PCT level. Although the aforementioned study^12^ has shown that there is no difference in PCT levels between patients with non-infected lung cancer and those with infection, another prospective study did not find that PCT levels in lung cancer were higher than those in healthy people, and only 2 of 79 patients had PCT levels above 0.5 μg/mL^13^.

In agreement with the findings of the present study, a large number of studies showed that dosimetric parameters of radiotherapy were related to the risk of radiation-induced lung injury^14,15^. We found that the patients treated with prescription dose higher than 62 Gy or mean lung dose higher than 17.4 Gy were more likely to suffer from ARP. Because of the small sample size we did not conduct further multivariate analysis, but we speculate that the combination of these dosimetric parameters of radiotherapy and PCT may further improve the accuracy of the ARP diagnosis. It deserves further investigation in a prospective study.

There are several limits in the present study that deserve discussion. First, in addition to BP, ARP also needs to be differentiated from other infectious pneumonia, such as pneumonia caused by fungi and viruses. Because previous studies have suggested that PCT is insensitive to these infectious pneumonias^16,17^, this study did not include these pneumonias. Because formal testing for viral pathogens was not performed in this study, we could neither exclude the possibility of bacterial/viral co-infection in the BP cohort or exclude the possibility of viral infection in the ARP cohort. Therefore, in the clinic we should use other examination methods for differential diagnosis. Second, the nature of retrospective studies precludes the researcher from the benefits of a prospective study. Such benefits include acquisition of specifically desired information and avoidance of the bias. Third, the sample size of this study is relatively small. Although the findings in this study are significant and confirm our hypothesis, a further prospective study with a large sample is necessary.

In conclusion, this study indicates that PCT is a useful biomarker for discriminating ARP from BP in lung cancer patients who have undergone thoracic radiotherapy.

## Data Availability

The datasets generated during and/or analyzed during the current study are available from the corresponding author on reasonable request.
